# Rapid, Simultaneous, and Automatic Determination of Lead and Cadmium in Cereals with a New High Performance Composite Hollow Cathode Lamp Coupled to Graphite Furnace Atomic Absorption Spectrometry

**DOI:** 10.3390/molecules27238571

**Published:** 2022-12-05

**Authors:** Yanxiang Wu, Songxue Wang, Weibing Cui, Wei Tian, Jieqiong Zhang, Xi Chen, Minghui Zhou

**Affiliations:** 1 The Academy of National Food and Strategic Reserves Administration, Beijing 100037, China; 2 Beijing Purkinje General Instrument Co., Ltd., Beijing 101299, China

**Keywords:** cadmium, lead, simultaneous determination, pretreatment method, automatic, lead-cadmium composite hollow cathode lamp, cereals

## Abstract

A simple, rapid, sensitive, accurate, and automatic graphite furnace atomic absorption spectrometry (GFAAS) method for detecting Cd and Pb in cereals is presented. This method enables the simultaneous determination of Cd and Pb in cereals with a pre-treatment method of diluted acid extraction and a high-performance lead–cadmium composite hollow-cathode lamp (LCC-HCL), and it realizes automatic determination from sample weighing to result output through an automatic diluted acid extraction system. Under the optimization, Pb and Cd in cereals were simultaneously and automatically detected in up to 240 measurements in 8 h. The LOD and LOQ of this method were 0.012 and 0.040 mg·kg^−1^ for Pb, and 0.0014 and 0.0047 mg·kg^−1^ for Cd, respectively. The results of the four certified reference materials were satisfied; there was no significant difference compared with the ICP-MS method according to a *t*-test, and the RSDs were less than 5% for Cd and Pb. The recoveries of naturally contaminated samples compared with the ICP-MS method were favorable, with 80–110% in eight laboratories. The developed method is rapid, low-cost, and highly automated and may be a good choice for grain quality discrimination and rapid analysis of Cd and Pb in different institutions.

## 1. Introduction

Cadmium (Cd) and lead (Pb) are toxic heavy metals, and the harmful effects on human health caused by Pb and Cd contamination are well known [1,2]. It has been reported that cereals are the main contributors to Cd and Pb exposure [3]. In developing countries, Pb and Cd pollution are serious [4,5,6]. Considering the high cost of inductively coupled plasma mass spectrometry (ICP-MS), graphite furnace atomic absorption spectrometry (GFAAS) is still the dominant analytical technique used for Pb and Cd analysis in cereals [7,8,9].

GFAAS with single-element analysis is time consuming [10], and the pretreatment operation is complicated. At present, because of the low sensitivity for Pb and the high sensitivity for Cd in GFAAS, the current range of Cd is narrow. To detect cereal samples with consistent contamination levels of Pb and Cd, it is difficult to achieve the simultaneous detection of two heavy metals with the same preprocessor [11,12,13].

Notably, for many years, extensive research has been devoted to multi-element simultaneous measurements based on the GFAAS [14,15]. The application of high-resolution continuous light source technology has been found to be the most successful, and a variety of devices are available [16,17,18]. However, these technologies have good applications in flame atomic absorption but are not applicable to the detection of low contents of Pb and Cd in cereals; therefore, it is necessary to develop the simultaneous determination of lead and cadmium in cereals using GFAAS.

Based on this situation, a new high-performance lead–cadmium composite hollow-cathode lamp (LCC-HCL) was designed in this study. High-performance hollow-cathode lamps were first reported by Sullivan and Walsh [19], which could enhance sensitivity by providing a stronger and more stable radiation intensity, thus improving spectral interference. Subsequently, Rensburg and Zeeman [20] developed a multi-element, high-intensity hollow-cathode lamp with selective modulation for the detection of gold, platinum, palladium, and rhodium, which has since been widely applied [21,22]. Yingqi and Yongzhang [23] designed a new type of high-performance hollow cathode lamp that used two separate discharges to markedly increase light output and minimize the self-absorption broadening of the spectral line, linearity, and sensitivity. Furthermore, Ke and Yingqi et al. used this lamp to measure Pb in water, and the results indicated that the most sensitive analytical line Pb 217.0 nm exhibited better performance of stability, improved signal-to-noise ratio, and enhanced detection limit compared to the sub-sensitive analytical line Pb 283.3 nm [24,25]. J.B. Willis first reported that the copper (Cu) cathode could be used for the buckle background in the high-performance composite hollow cathode [26]; this method is for the background of non-resonant line buckles because it can provide more non-absorbed lines at a suitable wavelength [27].

In addition, traditional pretreatment methods require tedious steps, a large amount of strong corrosive reagents, and conditions of high pressure and temperature [28,29,30,31,32]. Therefore, they are time consuming and unfriendly to researchers and the environment, and it is difficult to realize automation. To overcome these disadvantages, we developed a rapid and green pretreatment method for diluted nitric acid extraction for the determination of Cd and Pb in cereals in previous studies [33,34,35,36], and it was shown to be a fast, convenient, and reliable pretreatment method that only uses 0.5% HNO_3_ as the extraction reagent. Furthermore, the diluted acid extraction technique is conducive to realizing automation.

In this study, a new GFAAS method was established with the development of the LCC-HCL. Through optimization of the conditions for diluted acid extraction, a fully automatic dilute acid extraction system was designed to realize the automated determination of Pb and Cd in cereals. This method is efficient, cost-effective, convenient, and suitable for rapid, simultaneous, and automated detection of Pb and Cd in cereals.

## 2. Results and Discussion

### 2.1. LCC-HCL Development and Evaluation

**Figure 1:**
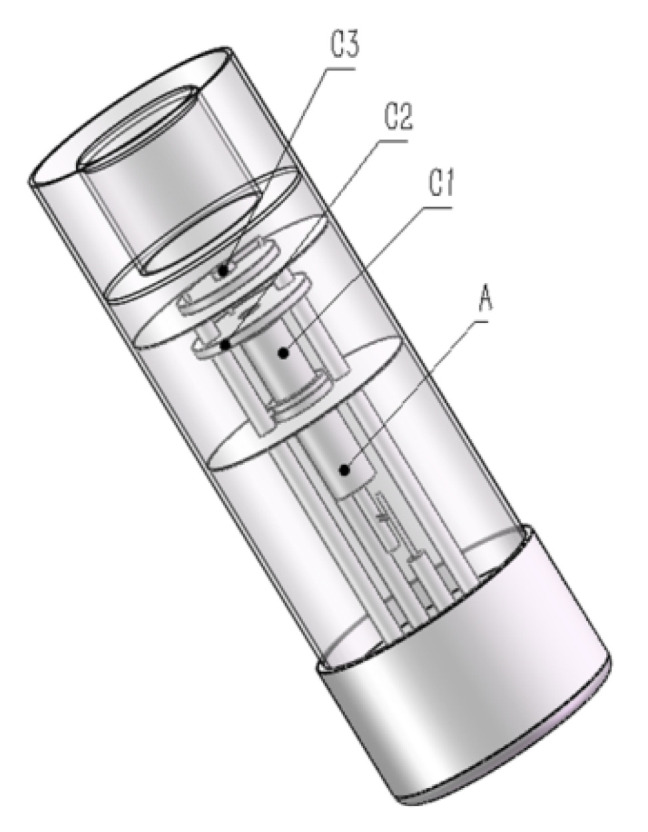
Pb and Cd high-performance composite hollow-cathode lamp. A: anode; C1: Pb–Cd composite cathode; C2: auxiliary cathode; C3: Cu cathode.

A hollow-cathode lamp was designed by combining a Pb–Cd composite cathode with a Cu cathode to buckle the background, as shown in Figure 1. The characteristic lines of 217.0 nm for Pb and 228.8 nm for Cd were adopted. The role of the auxiliary cathode is to cause a significant change in the intensity of the elements in the Pb–Cd composite cathode.

**Figure 2:**
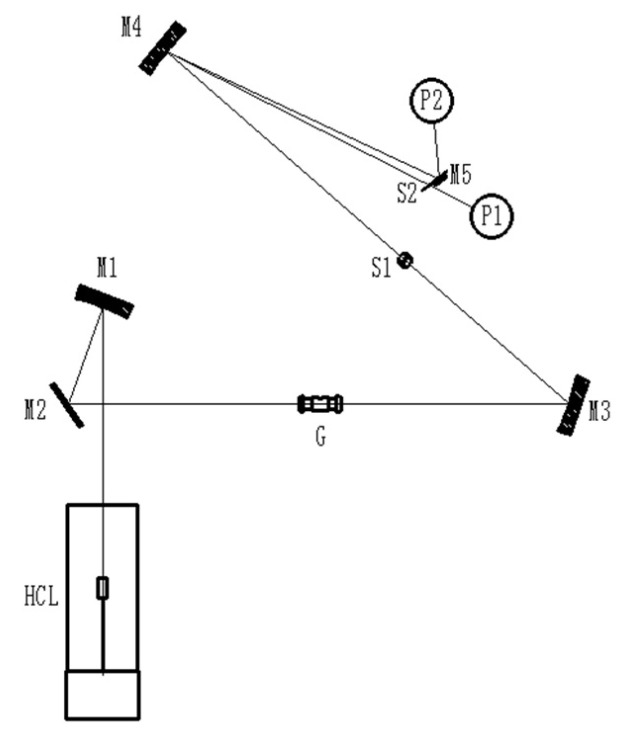
The optical path of atomic absorption spectrometry for Pb–Cd simultaneous determination. HCL: high-performance composite hollow-cathode lamp; M1–M3 and M5: mirrors; M4: concave grating; G: graphite tube; S1–S2: slit components; P1–P2: photomultiplier tubes.

A dual-channel simultaneous reception method was adopted to realize the reception and measurement of light at different wavelengths [37,38]. Figure 2 shows that the atomizer (G) was designed with a graphite cup with a large capacity, and the maximum injection volume reached 40 μL. The concave grating (M4) was used as a monochromator, the composite light was illuminated on the concave grating through the incident slit, and the characteristic spectral lines of Pb and Cd were imaged on the Rowland grating after being split. The 217.0 nm wavelength of Pb, 228.8 nm wavelength of Cd, and near wavelength of Cu were projected onto the position of the photomultiplier tubes (P1, P2) through the exit slit, and a reflector (M5) was added to reach the appropriate position. The construction ensured that light with different wavelengths was received and measured, and optical paths were successfully developed for the simultaneous determination of Pb and Cd.

To ensure that these high-performance composite hollow-cathode lamps could be used for the detection of Pb and Cd in cereals, lamp performance was investigated by testing the noise, drift, and sensitivity of three random composite hollow cathode lamps (Table 1). The results showed that the noise, drift, and consistency of the sensitivity met the requirements of the application.

### 2.2. Optimization of the Method

#### 2.2.1. Optimization of the Heating Program

The same heating program was used to detect Cd and Pb in cereals, as shown in Table 2. To achieve the best detection, the pyrolysis temperature and atomization temperature were optimized.

**Figure 3:**
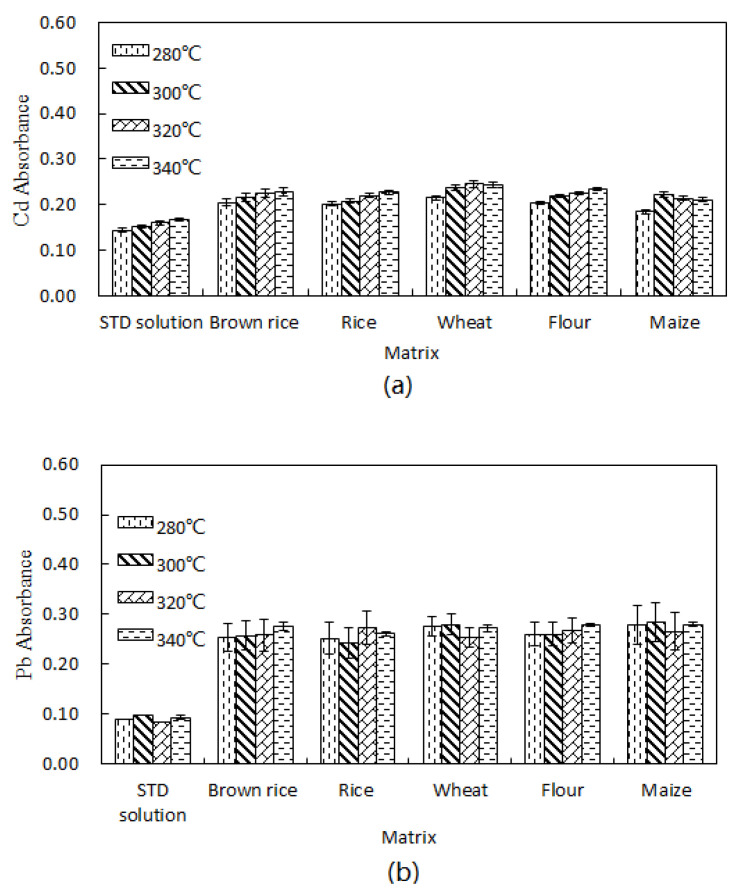
Effect of different pyrolysis temperatures on different grain matrices (n = 5): (**a**) Cd and (**b**) Pb.

The pyrolysis temperature was optimized by testing a standard solution (STD solution) and five cereal extractions (Figure 3). Considering the sensitivity and background, a temperature of 320 °C was selected as the optimum pyrolysis temperature. Similarly, the optimum atomization temperature was 1700 °C.

#### 2.2.2. Extraction of Pb and Cd from Cereals

Based on our previous study [33,34], a diluted nitric acid solution was selected to extract Pb and Cd from cereals. To ensure that the Pb and Cd in cereals were extracted completely under the same conditions, the key factors were optimized with three different cereals. The optimized results showed that the following: the concentration of nitric acid solution needed to be above 5%; the extraction temperature needed to be above 20 °C, which was room temperature; the particle diameter size of the samples needed to be less than 0.38 mm; the ratio of liquid to solid needed to be between 1:25 and 1:50; the extraction time needed to be at least 5 min; and it was necessary to separate the liquid and solid under the condition of a free setting time of 10 min. (The details of the optimization are described in the Supplementary Materials).

### 2.3. Automation Diluted Acid Extraction System

**Figure 4:**
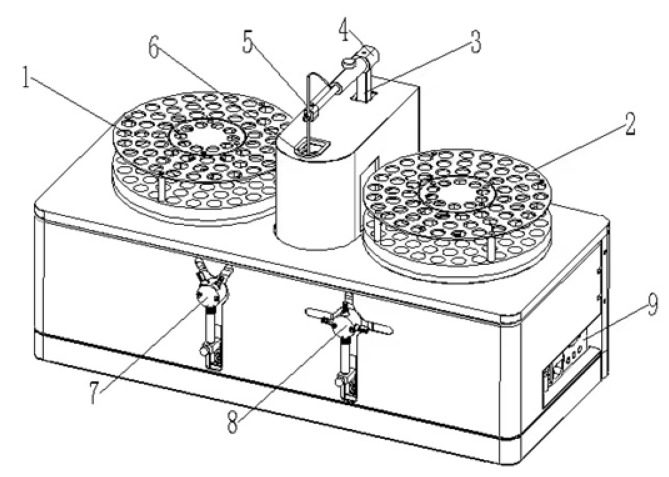
The automated diluted acid extraction system: (1) left sample tray with oscillation part; (2) right sample tray with oscillation part; (3,4) rotary lifting injection mechanical arm; (5) injection needle; (6) washing basin; (7) 5 mL syringe pump; (8) 100 μL syringe pump; (9) interface assembly board.

**Figure 5:**
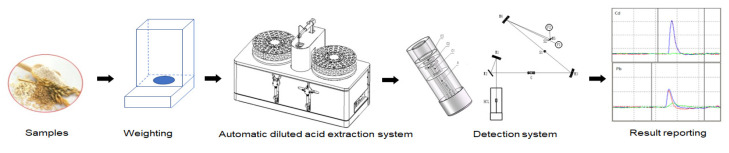
Flow chart of the simultaneous determination of Pb and Cd in cereals with an automated diluted acid extraction system.

To realize the automation of Pb and Cd detection, an automated diluted acid extraction system was designed, as shown in Figure 4, under the optimized conditions of the pre-treatment. The system includes a real-time reading part for balance, a part for adding the extraction solution, an oscillation part, and an automatic sample injection part, which can realize the automatic determination from extraction of the sample to the result output. The simultaneous and automated determination of Pb and Cd in cereals is shown in Figure 5. An electronic balance with a communication port is used to read the weighing quality of the sample in real time, and the effective traceability of the measurement results is achieved through software recording. A high-precision syringe pump was used to add the acid solution to the designated sample tube automatically. Magnetic stirring and a constant temperature heating plate were used to realize the oscillation for extraction (5 min). After the setting time (10 min), automatic sample injection and dilution were realized through the sample injection arm and the high-precision injection pump. The sample was then detected by a detection system to obtain the concentration of Pb and Cd in the sample (1.5 min). With this system, up to 240 measurements could be realized within 8 h.

### 2.4. Interferences

#### 2.4.1. Inorganic Interference

The 217.0 nm characteristic spectral line of Pb and the 228.8 nm characteristic spectral line of Cd may have inorganic interference, including the spectral interference of coexisting Fe and Sn and the coexisting element interference of Zn and Cu.

The structure of GFAAS with LCC-HCL avoids spectral interference, as only 217.0 nm and 228.8 nm of light can pass through the grating, as shown in Figure 2. To ensure that the 228.723 nm for Fe and 228.668 nm for Sn would not cause spectral interference of the 228.802 nm spectrum wavelength for Cd, 1 mg·L^−1^ Fe and Sn solution were added into different concentrations of standard solutions, and the coexisting element interference was studied by adding 1 mg·L^−1^ Zn and 1 mg·L^−1^ Cu solution into different concentrations of standard solutions, and series standard solutions without any additional addition of other elements were used for comparison. The results shown in Table 3 indicate that the relative difference between the measured and theoretical values of each group was not significantly different from the comparison; therefore, no inorganic interference was observed when GFAAS with LCC-HCL was employed to simultaneously detect Cd and Pb in cereals.

#### 2.4.2. Matrix Effect

The most difficult problem to solve is the matrix effect from some soluble proteins, sugars, and fats [39], which can affect the detection of Pb and Cd. To eliminate matrix interference, the chemical matrix modifier, standard curves of different matrices, and their corresponding results were investigated.

**Figure 6:**
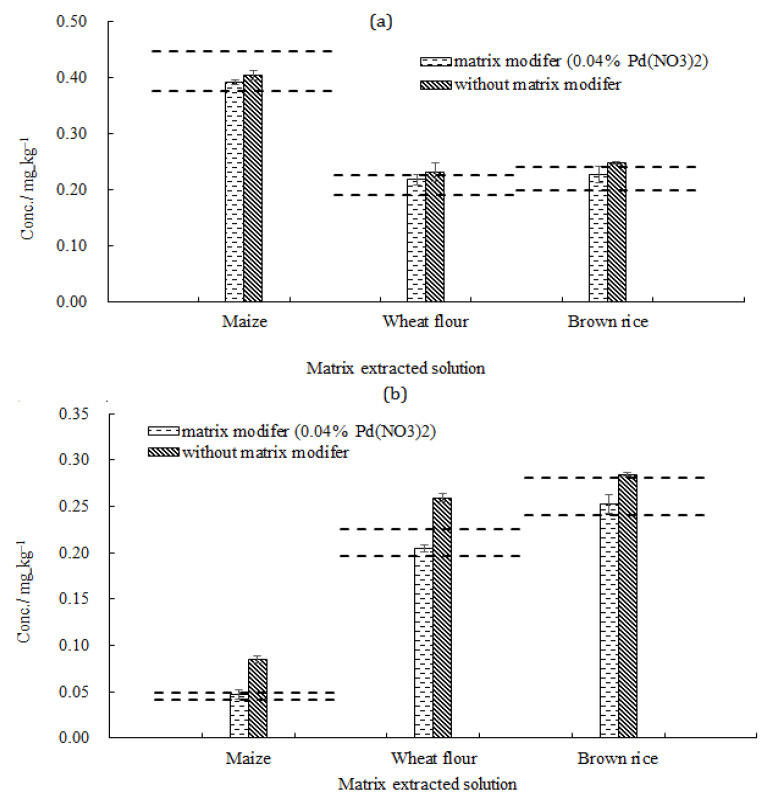
The influence of the matrix modifier in different matrix-extracted solutions (n = 3): (**a**) Pb; (**b**) Cd. The dotted line represents the expanded uncertainty of the certified value. *U_crm_ = k·u_crm_* (*k* = 2).

After optimization, 0.04% Pd(NO_3_)_2_ was selected to improve the determination of Cd and Pb in cereals. Based on the matrix matching standard curve, three matrix reference materials were used to conduct a comparative study with and without matrix modifiers, and the results are shown in Figure 6. It was shown that a chemical matrix modifier of 0.04% Pd(NO_3_)_2_ was necessary to obtain an accurate value.

**Figure 7:**
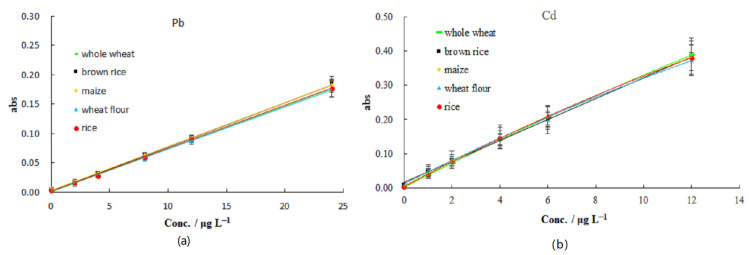
The different matrix matching standard curves (n = 5): (**a**) Pb and (**b**) Cd.

To further determine which should be selected for matrix matching, different matrix-matching standard calibration curves were established to eliminate matrix interference and obtain accurate values for Pb and Cd (Figure 7). The standard curves of the different matrices showed no significant differences, and three certified reference materials were applied. The corresponding detected values of these matrix matching standard curves are presented in Table 4, which shows that the detected values were all within the range of the certified reference material assignment. To sum up, it is sufficient to obtain accurate and reliable test results for Pb and Cd by using the matrix matching standard curve of any blank cereal and a matrix modifier of 0.04% Pd(NO_3_)_2_.

### 2.5. Performance of the Method

#### 2.5.1. Trueness

The trueness of the method was identified by comparison with the standard method (microwave digestion for pre-treatment and detection by ICP-MS), and four reference materials (maize flour, brown rice flour, wheat flour, and rice flour) as the matrix samples. The results summarized in Table 5 indicate that the value was within the certified value and uncertainty, and the results showed no statistical difference between the two methods at the 95% level according to the *t*-test, which indicates that the method is accurate enough for the simultaneous determination of Cd and Pb in cereals.

#### 2.5.2. Precision

The precision of the method was studied using a standard solution (Pb 8 μg·L^−1^, Cd 4 μg·L^−1^) and three matrix extracted solutions of maize, brown rice, and wheat. The results showed that the RSDs were 1.5% and 1.8% for Pb and Cd in the brown rice extracted solution, 4.4% and 2.9% for Pb and Cd in the wheat extracted solution, 3.2% and 4.8% for Pb and Cd in the maize extracted solution, and 2.2% and 2.7% for the Pb and Cd in the standard solution, respectively, indicating that there was no significant difference between the standard solution and the matrix sample extraction solution, and the RSDs based on seven measurements were always lower than 5.0%.

#### 2.5.3. Calibration Curves, Linearity, and Limit of Detection and Quantification

The calibration curves, linearity, and limit of detection and quantification are shown in Table 6. The limit of detection (LOD) and the limit of quantification (LOQ) were calculated using blank samples (the concentrations of Pb and Cd were both lower than 0.010 mg·kg^−1^) according to ISO 11843-5:2008 [40]. When the weight was 0.2 g and the extraction solution volume was 5 mL, the LOD and LOQ were calculated and expressed in mg·kg^−1^. The LOD of this method for Pb decreased in multiples compared with the traditional GFAAS [34], so the reliability and practicability were greatly enhanced.

### 2.6. Naturally Contaminated Sample Analysis

The method was further applied to the naturally contaminated samples analysis. Ten typical samples containing both Pb (0.090 mg·kg^−1^–0.40 mg·kg^−1^) and Cd (0.020 mg·kg^−1^–0.45 mg·kg^−1^) were chosen as the application samples, which included brown rice, wheat, and maize. The naturally contaminated samples were analyzed using the microwave digestion-ICP-MS method for comparison, and then analyzed using the optimized methods of this study in eight laboratories. The relative recoveries were calculated and are shown in Table 7 and Table 8. The relative recoveries were favorable at 80–117%, and the results of the different labs showed good consistency. It was proven that the method developed in this study has good availability, and it could be a satisfactory choice to determine different concentrations of Pb and Cd in various cereal matrices.

## 3. Materials and Methods

### 3.1. Chemicals and Testing Samples

HNO_3_ (Guaranteed Reagent, Merck, Kenilworth, NJ, USA), stock solutions of Pb, Cd, Zn, Cu, Fe, and Sn (1000 mg·L^−1^, The National Institute of Metrology of China, Beijing, China), and high-purity argon (99.999%, Beijing Qianxi Gas Cylinder Co., Ltd., Beijing, China) were used. All solutions were prepared with ultrapure water (18.2 MΩ.cm, Milli-Q). Different concentrations of Cd and Pb solutions, 1 mg·L^−1^, and Cu, Fe, and Sn solutions were diluted with HNO_3_ solution (5%, *v*/*v*).

Samples (brown rice, wheat, and maize powder) containing Pb and Cd and blank samples (brown rice powder, rice powder, wheat powder, flour powder, and maize powder without Pb and Cd) were provided by the Academy of National Food and Strategic Reserves Administration. Certified reference materials (CRMs), including GBW (E) 080684a (rice flour), GBW (E) 100377 (brown rice flour), GBW100379 (wheat flour), GBW08503c (wheat flour), and GBW (E) 100380 (maize flour), were obtained from the National Institute of Metrology of China.

### 3.2. Instruments and Conditions

A Milli-Q purification system was used to provide the ultrapure water (18.2 MΩ·cm^−1^, EQ 7000, USA), and 0.15, 0.18, 0.25, 0.38, and 0.83 mm sample sieves (Ejiang, China) were used; analytical balance (Sartorius, BS224S, Niedersachsen, Germany); centrifuge (Sigma 3–30 K, Roedermark, Germany); grinding mill (Retsh SR300, Haan, Germany), the particle size was 60 meshes (0.250 mm); and microwave digestion system (Preekem Topex+, Shanghai, China).

An atomic absorption spectrometer (TAS 986, Purkinje, China) with a newly designed optical path (Figure 2) was used for the simultaneous determination of Pb and Cd. The LCC-HCL was manufactured according to our design (Figure 1) by being entrusted to a company with legal manufacturing qualifications (Baoding Hengming Light Source Electronic Technology Co. Ltd., Baoding, China). The operating conditions and heating program of GFAAS are listed in Table 2.

Inductively coupled plasma mass spectrometry (ICP-MS, Agilent, 7500cx, Santa Clara, CA, USA) was used for comparison. The operating conditions for the optimization of the experiment are shown in Table 9.

### 3.3. Sample Preparation

The samples were ground in a mill until they passed through the sample sieve and then mixed thoroughly.

Samples for microwave digestion pre-treatment were prepared as follows: cereal samples (0.2–0.3 g) were digested with HNO_3_ (Guaranteed Reagent, 7 mL) using a microwave digestion system (the microwave digestion program is presented in the Supplementary Materials). The digestion solution was heated (160 °C) until it remained at 1–2 mL. The solution was transferred and fixed at 25 mL using a volumetric flask with ultrapure water; the blank was produced using the same procedure.

Samples for extraction were prepared as follows: Samples (0.2 g) were mixed with 5 mL HNO_3_ solution (5%, *v*/*v*), shaken for 5 min, and stood for 10 min.

Matrix-matching solution: 4 g blank cereals (brown rice powder, rice powder, wheat powder, flour powder, and maize powder without Pb and Cd) were weighed, and 100 mL HNO_3_ solution (5%, *v*/*v*) was added, followed by shaking for 5 min at a temperature above 20 °C, and standing for 10 min.

Matrix-matching standard calibration curves: Mix and dilute Cd and Pb standard solution to 50 μg·L^−1^ for Cd and 100 μg·L^−1^ for Pb with matrix-matching solution, stepwise dilute the solution into concentrations of 1, 2, 4, 6, and 12 μg·L^−1^ for Cd and 2, 4, 8, 12, and 24 μg·L^−1^ for Pb with matrix-matching solution.

The noise test for LCC-HCL: Three LCC-HCL were randomly selected and preheated for 30 min under lamp working conditions (Table 2). The noise is the instantaneous noise (peak to peak) recorded in 15 min.

The drift test for LCC-HCL: Three LCC-HCL were randomly selected and preheated for 30 min under lamp working conditions (Table 2). The drift is the zero-drift recorded in 15 min.

The sensitivity test for LCC-HCL: Three LCC-HCL were randomly selected and preheated for 30 min under lamp working conditions (Table 2), the average absorbance of 7 times determinations of 5% (*v*/*v*) HNO_3_ solution is A_0_, and the average absorbance of 7 times determinations of 10 μg·L^−1^ standard solutions of Pb and Cd is A_1_(Cd) or A_1_(Pb), the sensitivity is the difference between A_1_ and A_0_.

## 4. Conclusions

In this study, a rapid, automatic, and simultaneous method and an automatic diluted acid extraction system for the detection of Pb and Cd in cereals were developed. Under optimal conditions, the method was confirmed to have satisfactory trueness and precision. Moreover, Pb and Cd in cereals could be simultaneously and automatically detected in up to 240 measurements in 8 h when the automatic detection cycle was running, which could greatly reduce both the analysis time and errors caused by manual operation. The developed method is in line with the need for the automatic and efficient detection of Pb and Cd in cereals, and further optimization of the process could make this method applicable to the detection of Pb and Cd in other products, such as milk powder, vegetables, or oils.

## Figures and Tables

**Table 1:** Performance test of LCC-HCL (n = 3).

Element	Test Item	Result	Requirement
Lead	Drif (Abs/15 min)	0.000 ± 0.004	≤±0.008
	Noise (Abs)	0.0010	≤0.006
	Sensitivity (Abs)	0.137 ± 0.007	≤10
Cadmium	Drif (Abs/15 min)	0.002 ± 0.004	≤±0.008
	Noise (Abs)	0.0026	≤0.006
	Sensitivity (Abs)	0.333 ± 0.030	≤10

**Table 2:** Instrumental operating conditions and heating program for the simultaneous determination of Cd and Pb.

Spectrometer Conditions			Heating Program				
	Cd	Pb	Step	Temperature (°C)	Ramp (s)	Hold (s)	Argon
Wavelength (nm)	228.8	217.0	Drying 1	75	5	2	ON
Bandpass (nm)	2.0	0.7	Drying 2	90	5	2	ON
Sample volume (μL)	12	12	Drying 3	110	10	2	ON
Modifier Volume (μL)	3	3	Drying 4	120	5	2	ON
Lamp current (mA)	4	4	Pyrolysis	320	5	5	ON
			Atomization	1700	2	1	OFF
			Cleaning	2450	1	1	ON

**Table 3:** Study the influence of inorganic interference.

Added Element	Item	Cd			Pb		
	Theoretical Value (μg·L^−1^)	2	4	8	4	8	16
Comparison without addition of other elements	Measured value (μg·L^−1^)	2.13	3.93	7.99	4.19	8.18	15.94
	Relative difference (%)	6.4	−1.8	−0.1	4.8	2.3	−0.4
Zn	Measured value (μg·L^−1^)	1.91	4.18	7.95	4.08	7.84	15.93
	Relative difference (%)	−4.3	4.5	−0.6	2	−2	−0.4
Cu	Measured value (μg·L^−1^)	2.01	4.11	8.17	4.01	8.16	15.72
	Relative difference (%)	0.6	2.8	2.2	0.3	2	−1.8
Fe	Measured value (μg·L^−1^)	2.09	4.19	8.04	--	--	--
	Relative difference (%)	4.7	4.7	0.5	--	--	--
Sn	Measured value (μg·L^−1^)	2.12	3.87	7.85	--	--	--
	Relative difference (%)	5.8	−3.3	−1.9	--	--	--

**Table 4:** Results of certified reference materials using different matrix matching standard curves (value ± SD, n = 3).

Item		Matrix for Matching Standard Curve					Certified Value	Uncertainty
		Whole Wheat	Wheat Flour	Maize	Brown Rice	Rice		
Cd (mg·kg^−1^)	Brown Rice	0.262 ± 0.018	0.266 ± 0.016	0.256 ± 0.012	0.262 ± 0.014	0.252 ± 0.014	0.261	0.020
	Wheat	0.162 ± 0.013	0.165 ± 0.010	0.159 ± 0.012	0.165 ± 0.008	0.162 ± 0.009	0.155	0.013
	Maize	0.043 ± 0.007	0.041 ± 0.002	0.042 ± 0.002	0.043 ± 0.006	0.042 ± 0.004	0.045	0.004
Pb (mg·kg^−1^)	Brown Rice	0.224 ± 0.012	0.228 ± 0.012	0.222 ± 0.008	0.223 ± 0.016	0.226 ± 0.016	0.220	0.020
	Wheat	0.234 ± 0.013	0.238 ± 0.014	0.227 ± 0.010	0.226 ± 0.016	0.234 ± 0.015	0.220	0.018
	Maize	0.399 ± 0.016	0.408 ± 0.017	0.385 ± 0.008	0.387 ± 0.010	0.399 ± 0.016	0.417	0.030

**Table 5:** Trueness study of the method (n = 3).

Matrix	Certified No.	Analysis of Reference Materials				Comparison of Two Method	
		Detected Value		Certified Value Range		T Value (*p* > 0.05, t = 4.30)	
		(values ± SD, mg·kg^−1^)		(mg·kg^−1^)			
		Pb	Cd	Pb	Cd	Pb	Cd
Wheat	GBW(E)100379	0.207 ± 0.003	0.154 ± 0.006	0.202~0.238	0.142~0.168	0.32	1.02
Rice	GBW(E)080684a	0.208 ± 0.005	0.482 ± 0.009	0.205~0.245	0.454~0.510	1.78	2.89
Brown rice	GBW(E)100377	0.207 ± 0.007	0.257 ± 0.005	0.200~0.240	0.241~0.281	0.29	2.79
Maize	GBW(E)100380	0.401 ± 0.005	0.042 ± 0.002	0.387~0.447	0.041~0.049	1.24	2.67

**Table 6:** Calibration and limit of detection and quantification.

Item	Cd	Pb
Calibration curve equation	Abs = −0.00055 × [Conc.]^2^ + 0.0528 × [Conc.] + 0.0032	Abs = 0.0111 × Conc. + 0.0022
R^2^	0.999	0.999
LOD	0.0013 mg·kg^−1^ (0.051 μg·L^−1^)	0.012 mg·kg^−1^ (0.49 μg·L^−1^)
LOQ	0.0043 mg·kg^−1^ (0.17 μg·L^−1^)	0.040 mg·kg^−1^ (1.61 μg·L^−1^)

**Table 7:** Results of Pb by GFAAS with LCC-HCL in real samples (n = 3).

Sample	Matrix	Recovery of Pb in Different Labs (%)							
		1	2	3	4	5	6	7	8
1#	wheat	102.7	104.9	102.7	107.7	104.6	113.4	104.6	99.2
2#	wheat	102.8	101.8	97.3	101.8	108.7	101.3	108.7	92.9
3#	wheat	94.5	90.0	92.0	92.0	89.4	95.2	88.1	89.4
4#	maize	102.2	98.6	95.1	106.0	107.9	101.1	107.9	91.0
5#	maize	108.7	108.7	107.1	107.1	109.2	104.5	109.7	105.6
6#	maize	96.7	98.9	110.9	110.9	94.6	103.3	102.2	97.8
7#	brown rice	88.1	87.3	88.6	89.4	85.8	87.3	85.8	85.5
8#	brown rice	87.7	86.8	90.4	93.2	94.5	91.8	96.4	80.4
9#	brown rice	93.0	95.8	91.2	94.0	91.2	99.5	91.2	82.9

**Table 8:** Results of Cd by GFAAS with LCC-HCL in real samples (n = 3).

Sample	Matrix	Recovery of Cd in Different Labs (%)							
		1	2	3	4	5	6	7	8
1#	wheat	95.5	98.7	93.9	98.4	95.8	95.5	102.2	88.2
2#	wheat	94.8	104.6	105.5	97.2	101.3	95.6	103.8	93.5
3#	wheat	84.0	104.8	103.5	100.1	112.9	102.2	116.9	104.8
4#	maize	97.1	102.5	102.1	101.1	102.5	95.3	102.6	100.0
5#	maize	100.4	88.4	100.4	102.7	93.2	90.8	102.7	95.6
6#	maize	88.0	104.0	100.0	112.0	96.0	88.0	100.0	104.0
7#	brown rice	96.6	101.4	99.7	102.7	102.2	97.9	96.3	99.4
8#	brown rice	105.0	105.0	111.1	106.5	113.6	104.5	116.7	100.9
9#	brown rice	99.2	97.1	112.4	104.8	114.5	98.5	111.7	107.5

**Table 9:** Instrumental operating conditions for ICP-MS.

Item	Parameters	Item	Parameters
Analyzed mass	^111^Cd and ^208^Pb	Sampling depth	8.0 mm
RF power	1500 W	Torch-H	0.3 mm
Carrier gas flow rate	0.76 L min^−1^	Torch-V	0.4 mm
Makeup gas flow rate	0.45 L min^−1^	Integration time	0.3 s·point^−1^
Nebulizer pump flow rate	0.10 rps	Interference equation	[^208^Pb] = [206] + [207] + [208]

## Data Availability

The data presented in this study are available on request from the corresponding author.

## Supplementary Materials

The following supporting information can be downloaded at: https://www.mdpi.com/article/10.3390/molecules27238571/s1, Table S1: Microwave digestion program; Table S2: Effect of extraction time for Cd and Pb; Table S3: The effect of the setting mode and time for Cd and Pb; Figure S1: Influence of different acid concentration (n = 3): (a) Cd and (b) Pb; Figure S2: Influence of different grain diameter (n = 3): (a) Cd and (b)Pb; Figure S3: Influence of the ratio of solid to liquid (n = 3): (a) Cd and (b) Pb; Figure S4: Influence of the extraction temperature (n = 3): (a) Pb in brown rice, (b) Pb in wheat, (c) Pb in maize, (A) Cd in brown rice, (B) Cd in wheat, and (C) Cd in maize, the dotted line represents the expanded uncertainty of the certified value, *U_crm_ = k·u_crm_* (k = 2).

## Sample Availability

Samples of the compounds are not available from the authors.

## References

[1] Ma C., Iwaishimada M., Tatsuta N., Nakai K., Isobe T., Takagi M., Nishihama Y., Nakayama S.F. (2020). Health Risk Assessment and Source Apportionment of Mercury, Lead, Cadmium, Selenium, and Manganese in Japanese Women: An Adjunct Study to the Japan Environment and Children’s Study. Int. J. Environ. Res. Public Health.

[2] Petrini R., Ghezzi L., Arrighi S., Genovesi L., Frassi C., Pandolfi L. (2022). Trace Elements in Soil and Urban Groundwater in an Area Impacted by Metallurgical Activity: Health Risk Assessment in the Historical Barga Municipality (Tuscany, Italy). Int. J. Environ. Res. Public Health.

[3] Rahman M., Islam M.A., Khan R.A. (2018). Characterization of chemical elements in common spices of Bangladesh for dietary intake and possible health risk assessment by INAA and AAS techniques. J. Radioanal. Nucl. Chem..

[4] Javaid S., Ashraf K., Sultan K., Siddiqui M.H., Ali H.M., Chen Y., Zaman Q.U. (2022). Risk Assessment of Potentially Toxic Metals and Metalloids in Soil, Water and Plant Continuum of Fragrant Rice. Agronomy.

[5] Weerasundara L., Magana-Arachchi D.N., Ziyath A.M., Goonetilleke A., Vithanage M. (2018). Health risk assessment of heavy metals in atmospheric deposition in a congested city environment in a developing country: Kandy City, Sri Lanka. J. Environ. Manag..

[6] Zhan L.B., Fu C., Wang Z.Y., Qi F.Y., Shao Z.L., Jing M. (2018). Heavy Metal Contamination and Health Risk Assessment in the Soil Surrounding a Secondary Lead Plant. Environ. Sci. Technol..

[7] Pacer E.J., Palmer C.D., Parsons P.J. (2022). Determination of lead in blood by graphite furnace atomic absorption spectrometry with Zeeman background correction: Improving a well-established method to support a lower blood lead reference value for children. Spectrochim. Acta Part B At. Spectrosc..

[8] Ataee M., Ahmadi-Jouibari T., Fattahi N. (2016). Application of microwave-assisted dispersive liquid–liquid microextraction and graphite furnace atomic absorption spectrometry for ultra-trace determination of lead and cadmium in cereals and agricultural products. Int. J. Environ. Anal. Chem..

[9] De Oliveira R.M., Antunes A.C.N., Vieira M.A., Medina A.L., Ribeiro A.S. (2016). Evaluation of sample preparation methods for the determination of As, Cd, Pb, and Se in rice samples by GF AAS. Microchem. J..

[10] Akinyele I.O., Shokunbi O.S. (2015). Comparative analysis of dry ashing and wet digestion methods for the determination of trace and heavy metals in food samples. Food Chem..

[11] Silvestre D.M., Nomura C.S. (2013). Direct Determination of Potentially Toxic Elements in Rice by SS-GF AAS: Development of Methods and Applications. J. Agric. Food Chem..

[12] Qi Y., Qi Z., Yang H., Zhang T. (2019). Uncertainty evaluation of determination of cadmium in rice by graphite furnace atomic absorption spectrometry. J. Food Safety Qual..

[13] Luo H., Ke S., Yan Q., Li W. (2015). The Comparison of Three Methods for the Detection of Cadmium in the Environment. Environ. Sci. Technol..

[14] Welz B., Vale M.G.R., Pereira É.R., Castilho I.N.B., Dessuy M.B. (2014). Continuum source atomic absorption spectrometry: Past, present and future aspects-a critical review. J. Braz. Chem. Soc..

[15] Leite C.C., de Jesus A., Potes M.L., Vieira M.A., Samios D., Silva M.M. (2015). Direct Determination of Cd, Co, Cu, Fe, Mn, Na, Ni, Pb, and Zn in Ethanol Fuel by High-Resolution Continuum Source Flame Atomic Absorption Spectrometry. Energy Fuels.

[16] Welz B., Becker-Ross H., Florek S., Heitmann U. (2006). High-Resolution Continuum Source AAS: The Better Way to Do Atomic Absorption Spectrometry.

[17] Ferreira S.L.C., Bezerra M.A., Santos A.S., Santos W.N.L.d., Novaes C.G., de Oliveira O.M.C., Oliveira M.L., Garcia R.L. (2018). Atomic absorption spectrometry—A multi element technique. Trends Anal. Chem..

[18] Aleluia A.C.M., de Santana F.A., Brandao G.C., Ferreira S.L.C. (2017). Sequential determination of cadmium and lead in organic pharmaceutical formulations using high-resolution continuum source graphite furnace atomic absorption spectrometry. Microchem. J..

[19] Sullivan J.V., Walsh A. (1965). High intensity hollow-cathode lamps. Spectrochim. Acta.

[20] Rensburg H.C.V., Zeeman P.B. (1968). The determination of gold, platinum, palladium and rhodium by atomic absorption spectrophotometry with an ultrasonic nebulizer and a multi-element high-intensity hollow-cathode lamp with selective modulation. Anal. Chim. Acta.

[21] Myöhänen T., Mäntylahti V., Koivunen K., Matilainen R. (2002). Simultaneous determination of As, Cd, Cr and Pb in aqua regia digests of soils and sediments using electrothermal atomic absorption spectrometry and fast furnace programs. Spectrochim. Acta B At. Spectrosc..

[22] Ferreira H.S., Santos A.C., Portugal L.A., Costa A.C., Miró M., Ferreira S.L. (2008). Pre-concentration procedure for determination of copper and zinc in food samples by sequential multi-element flame atomic absorption spectrometry. Talanta.

[23] Gao Y., Zheng Y. (1991). High Performance Hollow Cathode Lamp. Mod. Sci. Instrum..

[24] Othman M.H.D., Droushiotis N., Wu Z., Kelsall G., Li K. (2011). High-Performance, Anode-Supported, Microtubular SOFC Prepared from Single-Step-Fabricated, Dual-Layer Hollow Fibers. Adv. Mater..

[25] Chi K., Gao Y. (1994). Determination of Lead in Water by Microflame Atomic Absorption Spectrometry. Chin. Anal. Lab..

[26] Bakırdere S., Yaroğlu T., Tırık N., Demiröz M., Fidan A.K., Maruldalı O., Karaca A. (2012). Determination of As, Cd, and Pb in Tap Water and Bottled Water Samples by Using Optimized GFAAS System with Pd-Mg and Ni as Matrix Modifiers. J. Spectrosc..

[27] Kas R., Hummadi K.K., Kortlever R., De Wit P., Milbrat A., Luiten-Olieman M.W., Benes N.E., Koper M.T.M., Mul G. (2016). Three-dimensional porous hollow fibre copper electrodes for efficient and high-rate electrochemical carbon dioxide reduction. Nat. Commun..

[28] (2003). Foodstuffs Determination of Trace Elements Determination of Lead, Cadmium, Zinc, Copper, Iron and Chromium by Atomic Absorption Spectrometry (AAS) after Dry Ashing.

[29] (2003). Foodstuffs Determination of Trace Elements Determination of Lead, Cadmium, Chromium and Molybdenum by Graphite Furnace Atomic Absorption Spectrometry (GFAAS) after Pressure Digestion.

[30] (2003). Foodstuffs Determination of Trace Elements Determination of Lead, Cadmium, Zinc, Copper and Iron by Atomic Absorption Spectrometry (AAS) after Microwave Digestion.

[31] AOAC Official Method 999.10 (2011). Lead, Cadmium, Zinc, Copper, Andiron in Foods Atomic Absorption Spectrophotometry after Microwave Digestion.

[32] (2017). Animal and Vegetable Fats and Oils. Determination of Cadmium Content by Direct Graphite Furnace Atomic Absorption Spectrometry.

[33] Zhou M.H., Wang S.X., Wu Y.X. (2014). Rapid Direct Sampling Detection of Pb in Grain Using Diluted Acid Extraction Coupled with Graphite Furnace Atomic Absorption Spectrophotometry. Chin. J. Anal. Chem..

[34] Zhou M.H., Wu Y.X., Zhang J.Q., Zhang Y., Chen X., Ye J., Wang S.X. (2019). Development and Collaborative Study of a Diluted Acid Mild Extraction Method for Determination of Cadmium in Grain by Graphite Furnace Atomic Absorption Spectrometry. Anal. Sci..

[35] Zhang J.Q., Zhou M.H., Tian W., Wu Y.X., Chen X., Wang S.X. (2019). In situ fast analysis of cadmium in rice by diluted acid extraction-anodic stripping voltammetry. RSC Adv..

[36] Zhou H.M., Tian W., Zhang Q.J., Chen X., Wu X.Y., Wang X.S. (2019). A rapid on-site analysis method for the simultaneous extraction and determination of Pb^2+^ and Cd^2+^ in cereals. RSC Adv..

[37] Heitmann U., Welz B., Borges D.L., Lepri F.G. (2007). Feasibility of peak volume, side pixel and multiple peak registration in high-resolution continuum source atomic absorption spectrometry. Spectrochim. Acta B At. Spectrosc..

[38] Resano M., Aramendia M., Belarra M.A. (2014). High-resolution continuum source graphite furnace atomic absorption spectrometry for direct analysis of solid samples and complex materials: A tutorial review. J. Anal. Atom. Spectrom..

[39] Aljerf L., Mashlah A. (2017). Characterization and validation of candidate reference methods for the determination of calcium and magnesium in biological fluids. Microchem. J..

[40] (2008). Capability of Detection—Part 5: Methodology in the Linear and Non-Linear Calibration Cases.

