# Supplementary material for: Rapid, Simultaneous, and Automatic Determination of Lead and Cadmium in Cereals with a New High Performance Composite Hollow Cathode Lamp Coupled to Graphite Furnace Atomic Absorption Spectrometry
Source: Molecules. 2022 Dec 5;27(23):8571. doi: 10.3390/molecules27238571 (PMC9740317; doi:10.3390/molecules27238571)
Supplement: Supplementary file 1 [file molecules-27-08571-s001.zip › molecules-2040097-supplementary.pdf]

1 Microwave digestion program

The Microwave digestion program was given in Table S1.

Table S1 Microwave digestion program

Step	Temperature/℃	Keep time/min
1	80	3
2	120	3
3	150	3
4	180	20
5	200	25

2 Optimization of key factors for extracting Pb and Cd from grain samples using diluted acid

2.1 Optimization of the extraction concentration

According to previous study[39-40], diluted nitric acid solution was selected to extract Pb and Cd from grain samples, and the concentration was a key factor influencing the extraction efficiency. The optimization result of the extraction concentration was presented in Figure S1. To ensure the both extraction efficiency of Pb and Cd, the concentration need to be above 5% in three matrices, thus the concentration of 5% was finally adopted as optimal value.

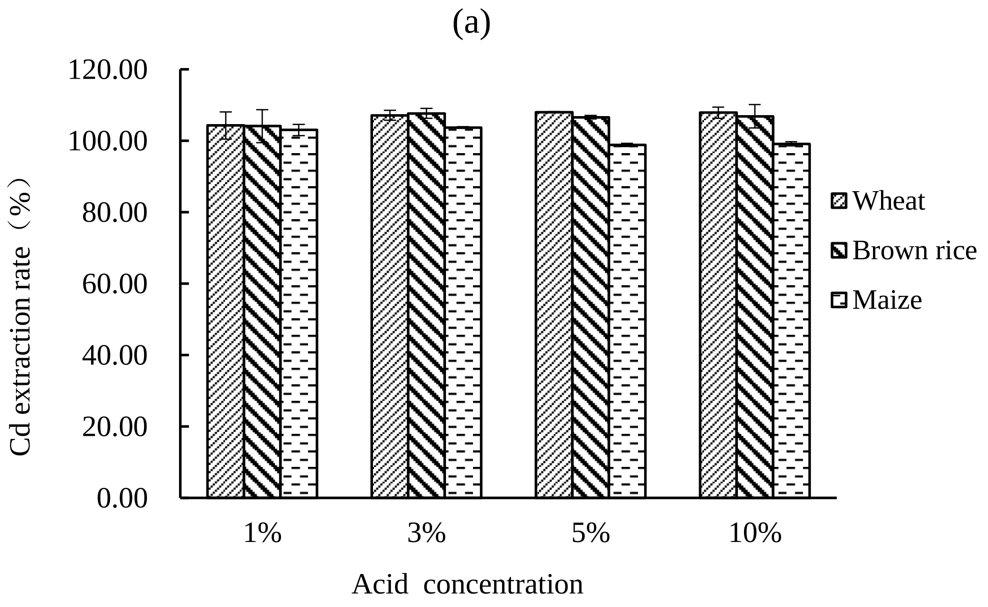

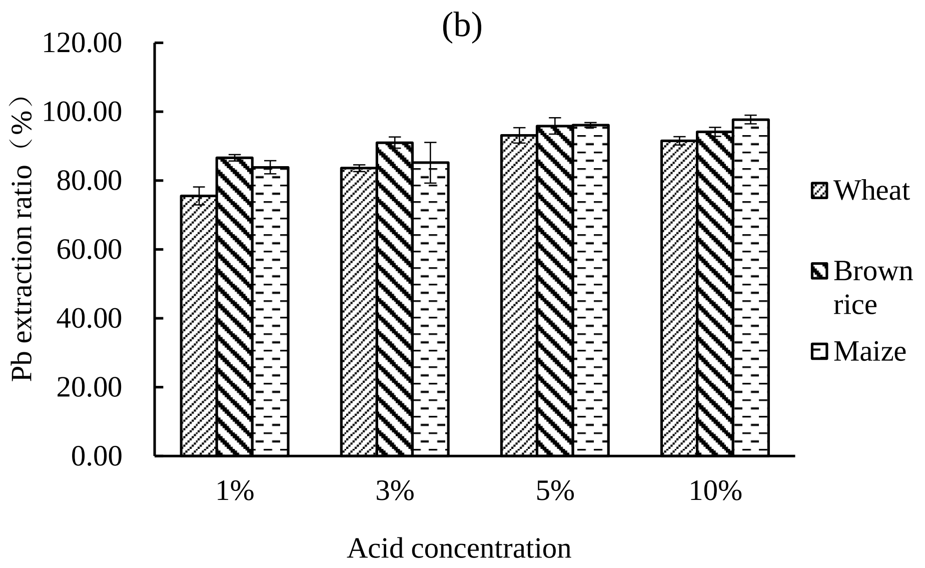

Figure S1. Influence of different acid concentration (n=3): (a) Cd and (b)Pb.

2.2 Optimization of extraction time

To ensure simultaneously thoroughly extraction of Pb and Cd from the grain samples, it was important to evaluate the effect of the extraction time. Table S2 summarizes that Cd did not exhibit significant change when the extraction time was set from 1 to 10 min. However, for three types of matrices, in particular maize, Pb needs to be immersed for at least 5 min to achieve an extraction rate of more than 90%. Therefore, the extraction of Pb and Cd from grain samples requires sufficient shaking for 5 min.

Table S2 Effect of extraction time for Cd and Pb

Extraction time	Pb, μg kg^{-1}			Cd, μg kg^{-1}		
	Maize	Brown rice	Wheat	Maize	Brown rice	Wheat
1 min	303	183	181	44	268	154
3 min	337	202	184	42	273	145
5 min	423	215	207	46	273	153
10 min	421	215	210	46	270	152
Certified Value	417	220	220	45	261	155
Uncertainty	30	20	18	4	20	13

2.3 Optimization of the setting time

Sample powders would settle down soon over time after shaking for 5 min, the result (Table S3) showed a free setting time of 10 min was enough.

Table S3 The effect of the setting time for Cd and Pb

Concentration, μg kg^{-1}		Pb, μg kg^{-1}			Cd, μg kg^{-1}		
		Rice	Wheat	Maize	Rice	Wheat	Maize
Free setting	5min	187	190	406	425	153	43
	10 min	225	214	411	456	155	42
Certified Value		226	220	417	482	155	45
Uncertainty		19	18	30	28	13	4

2.4 Optimization of the grain diameter

The wheat, maize, and brown rice samples were ground using five sieves with size of 0.83, 0.38, 0.25, 0.18, and 0.15 mm, respectively. The result is shown in Fig. S2, which demonstrates that Pb and Cd in the grain could be fully extracted when the particle diameter size of samples was less than 0.38 mm.

(a)

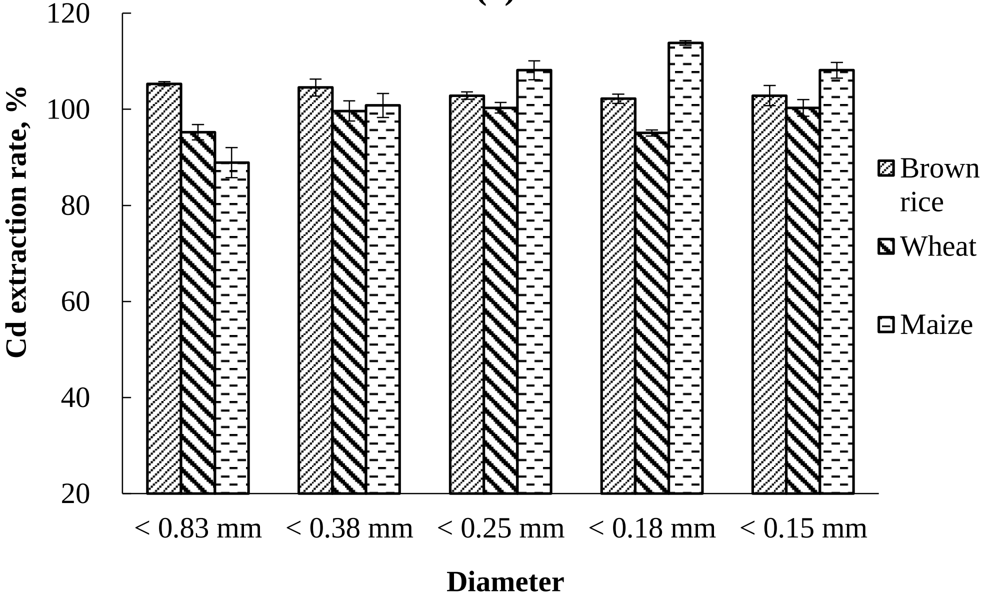

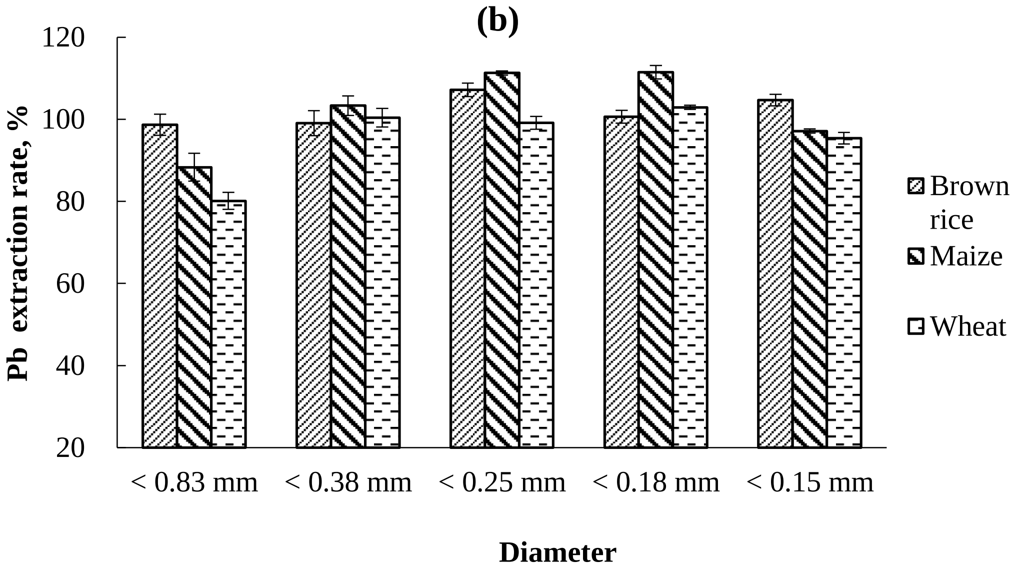

Figure S2. Influence of different grain diameter (n=3): (a) Cd and (b)Pb

2.5 Optimization of the ratio of liquid to solid

The ratio of liquid to solid may affect the extraction efficiency of Cd present in the grain sample. For convenient and sufficient shaking, the solid should be no more than 0.5 g, when the extraction solution was 5 mL. The final result indicated the occurrence of a good extraction and lower background absorbance when the ratio of liquid to solid was between 1:25 and 1:50 (Figure S3).

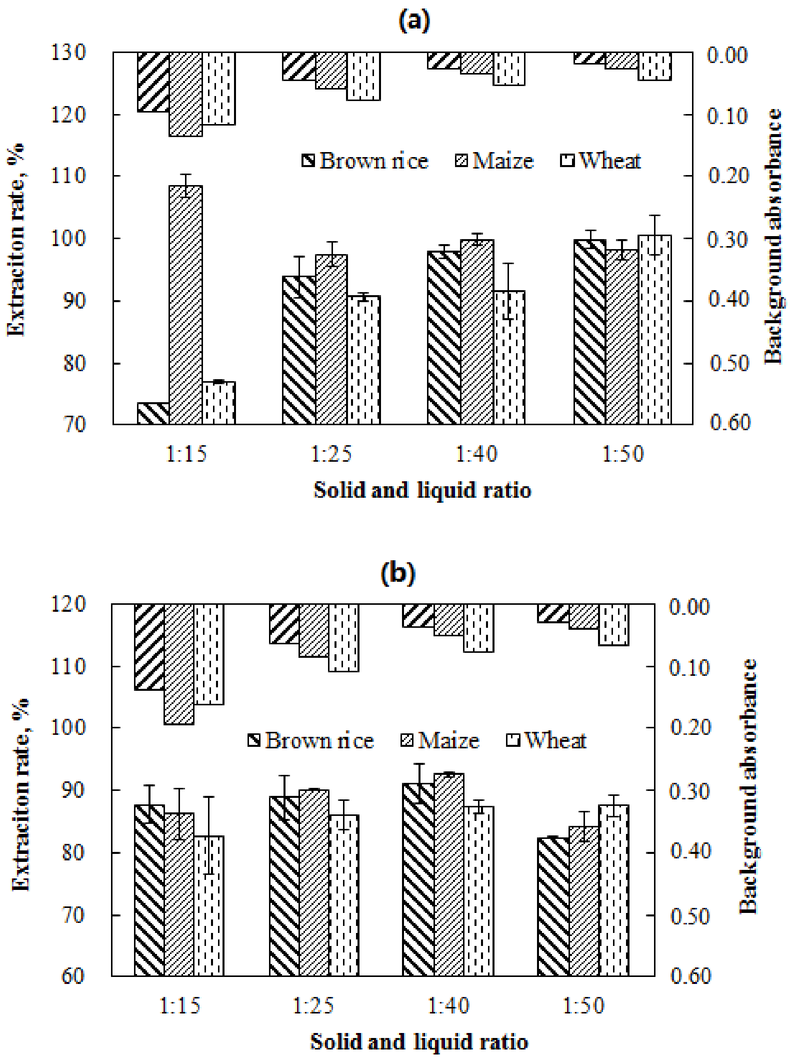

Figure S3. Influence of the ratio of solid to liquid (n=3): (a) Cd and (b) Pb

2.6 Optimization of the extraction temperature

Extraction temperature is an important factor affecting the extraction efficiency. For the sufficient extraction, the extraction temperature needs to be above 20°C to guarantee the extraction efficiency (Figure S4).

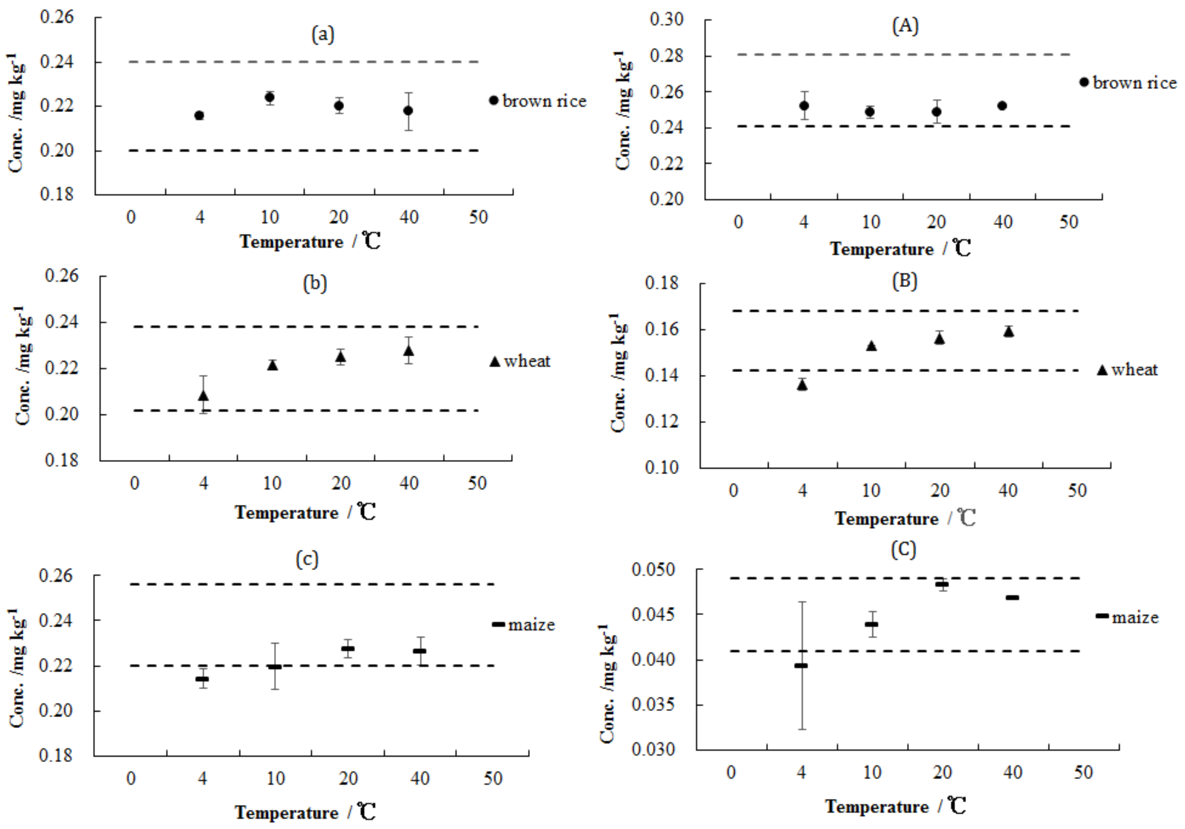

Figure S4. Influence of the extraction temperature (n=3): (a) Pb in brown rice, (b) Pb in wheat, (c) Pb in maize, (A) Cd in brown rice, (B) Cd in wheat, and (C) Cd in maize, the dotted line represents the expanded uncertainty of the certified value. $U_{crm}=k\,u_{crm}$ (k=2).
